# Guided neural style transfer for shape stylization

**DOI:** 10.1371/journal.pone.0233489

**Published:** 2020-06-04

**Authors:** Gantugs Atarsaikhan, Brian Kenji Iwana, Seiichi Uchida

**Affiliations:** Department of Advanced Information Technology, Kyushu University, Fukuoka, Japan; Aarhus University, DENMARK

## Abstract

Designing logos, typefaces, and other decorated shapes can require professional skills. In this paper, we aim to produce new and unique decorated shapes by stylizing ordinary shapes with machine learning. Specifically, we combined parametric and non-parametric neural style transfer algorithms to transfer both local and global features. Furthermore, we introduced a distance-based guiding to the neural style transfer process, so that only the foreground shape will be decorated. Lastly, qualitative evaluation and ablation studies are provided to demonstrate the usefulness of the proposed method.

## Introduction

**Fig 1:**
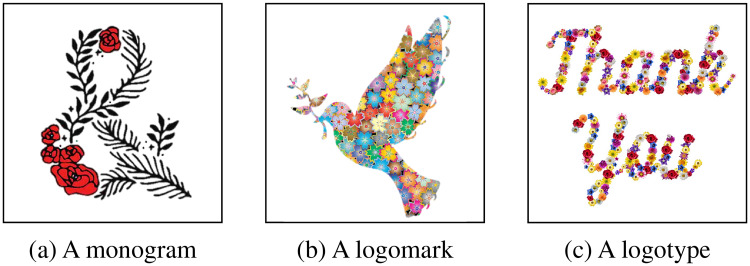
Examples of decorated shapes.

Designing decorated shapes (e.g. logos and typefaces) can require professional skills and can be time-consuming. Fig 1 shows examples of manually decorated shapes, such as a monogram, logomark, and logotype. Designers have to follow many complicated steps of designing processes, such as sketching and digital vectoring. Therefore, a method for automatically generating decorated shapes would be an important tool for both professional designers and non-professionals.

For logos, there exist applications, such as online tools [1, 2], that can be used for aided design. These tools generate logos by letting users choose from heuristic choices. However, due to the limited choice of fonts, shapes, and design patterns, there is the possibility for duplicate logos when two or more users unintentionally choose the same heuristic choices. Azadi et al. [3] and Yang et al. [4, 5] tried to generate stylized fonts and texts. Yet, these methods require prior training of the model for specific styles and fonts.

**Fig 2:**
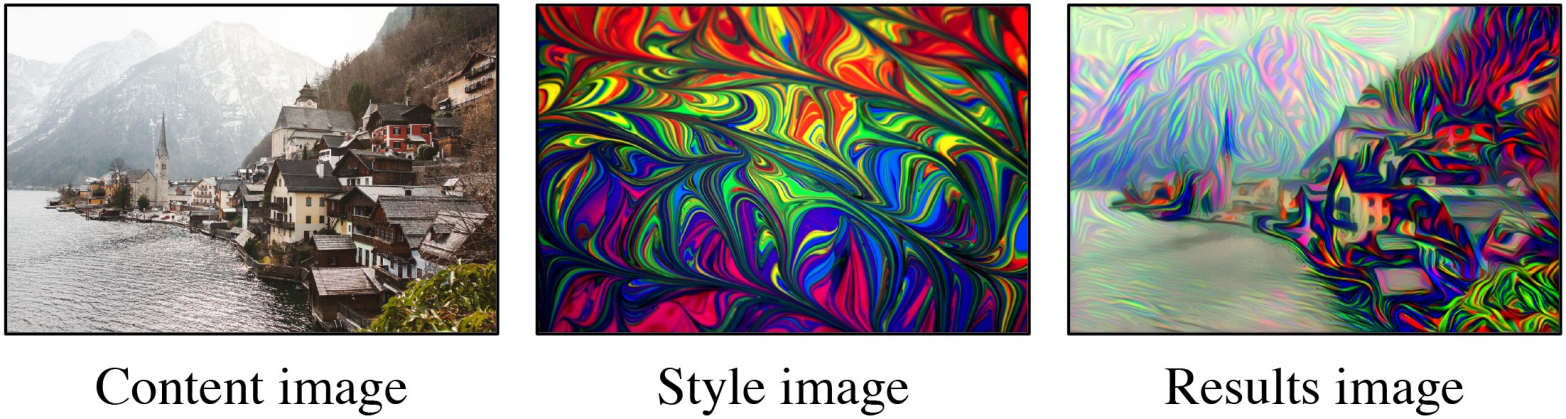
An example of NST. Features of the style image are blended into the structure of the content image in the results image.

In recent years, many studies have been conducted in the field of style transfer, which is the process of transferring styles from one image to another in order to generate a new stylized image. Recently, Gatys et al. [6] introduced the Neural Style Transfer (NST) algorithm for image style transfer using Convolutional Neural Networks (CNN) [7]. As shown in Fig 2, a new stylized image is created by synthesizing a *content* image and a *style* image. Specifically, an image is iteratively optimized from an initial image to that of an image with mixed qualities from the content image and the style image. The initial image can be anything; it could be a random image or the content image. The NST has been used in many domains, such as for fashion [8], portraits [9], photorealistic scenes [10, 11] and video style transfer [12].

The original NST method iteratively optimizes the initial image with a summary statistic, a Gram matrix. In this approach, global features are summarized into Gram matrices on specified layers of the CNN. However, the Gram matrix can be unstable because different sets of global features can be encoded into the same Gram matrix [13]. Furthermore, the Gram matrix ignores local features and their coherence. For instance, in Fig 2, the global features of the style image are densely populated multiple colors. With NST methods, the colors and density of the style image are transferred, and some local features of the style image (e.g. curved borders between the colors) are ignored. On the other hand, Li and Wand [14] proposed to capture local features by matching the patches from the feature maps in their Convolutional Neural Network Markov Random Fields (CNNMRF). In this approach, patches are extracted from feature maps, and directly matched to ensure that local features will not be ignored. The biggest disadvantage of the CNNMRF method is that it tends to transfer exact pixels of the style image. Because of that, the CNNMRF method produces nice and well-formed results only when global features of the content and style images are similar.

**Fig 3:**
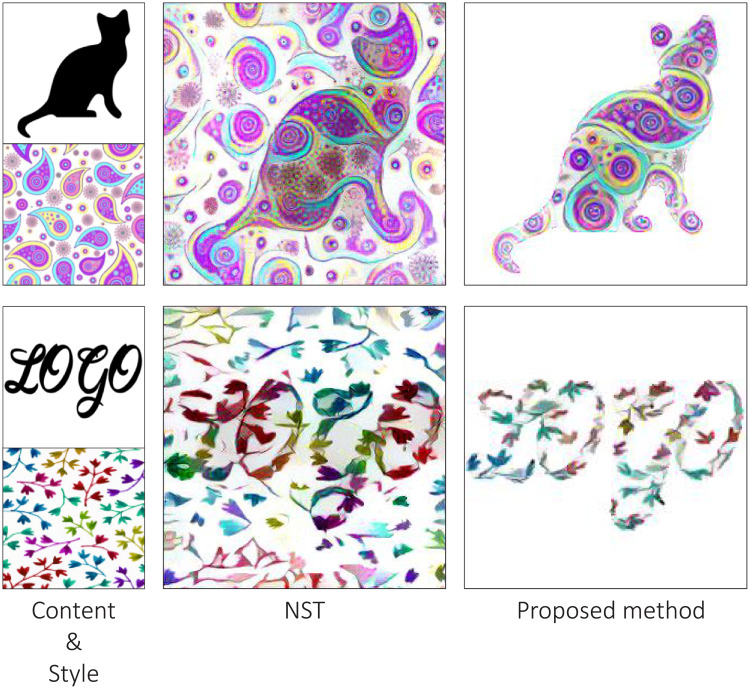
Examples of shape stylization. Compared to the NST, the proposed method was able to transfer the styles only to the foreground shape of the content image.

In this paper, we exploited the advantages of both parametric and non-parametric neural style transfer methods for stylizing images automatically. By combining these methods, we were able to transfer both correlations of global features and local features of the style image onto the content image simultaneously. However, the direct application of this method results in images that have styles in the foreground and background alike. Therefore, we constrained the style transfer process only to the areas in or near the foreground object of the content image with [15]. Some comparisons using the NST method are shown in Fig 3.

Furthermore, the proposed method does not require any prior training for specific styles in contrast to [3, 5]. Therefore, even people without any professional designing skills can create their unique results using any image as content or style image. Content image is restricted to clip arts and binary silhouette images as required in [15], while style image can be arbitrary. Due to that, the number of designs that can be created using the proposed method is limitless in contrast to the heuristic methods.

The main contributions of this paper are as follows:

- The first development of a neural style transfer-based shape design method.
- Suggested the combination of parametric and non-parametric neural style transfer methods into one model.
- Demonstrated a method of generating new and unique shapes (e.g., logos) easily.

## Related work

### Decorated shape generation

A classical approach to logo generation is the use of genetic algorithms. For example, Mark Maker [16] continuously generates logos based on other logos selected by the user. Each user-selected logo contributes to the seed for future generations. This genetic selection process continues until users find their desired logo. The problem with this approach is that no new logos are created; it combines user-defined text with existing shapes and attempts to guess the user’s preferred combination. Another problem is the possibility of users finding the same logo due to the limited choices. A more recent approach in logo generation is RepulsionPak by Saputra et al. [17]. In RepulsionPak, a container shape is filled with smaller shape elements iteratively. However, the smaller shape elements must be prepared for manipulation beforehand.

By using a CNN, Sage et al. [18] used Generative Adversarial Networks (GAN) [19] to generate logos. They gathered various logos from the Internet to create the Large Logo Dataset (LLD) and trained a GAN with the LLD to synthesize new logos. However, the logo synthesizing process was difficult to control. Therefore, Mino et al. [20] proposed to control the color of the resulting logos. Also, Atarsaikhan et al. [15] generated new logos from any shapes by constraining the original NST [6].

Regarding the creation of font images, Tsuchiya et al. [21] and Suveeranont et al. [22] proposed methods to use example fonts to determine the predictive features. Campbell et al. [23] and Uchida et al. [24] generated fonts using the interpolation of different fonts. More recently, fonts have been generated using NST [25] and GANs [26–28]. Furthermore, fonts have been stylized by statistical methods [4, 5] and by using GANs [3, 29, 30]. However, most of these methods require prior training for the style, making them difficult to use.

### Neural style transfer

Style transfer itself is not a new field. Hertzmann et al. [31] first introduced the example-based style transfer method. More recently, Gatys et al. opened a new field called, “Neural Style Transfer” [6]. Since then, many improvements and inspired methods introduced. According to Jing et al. [32], neural style transfer field is divided into image optimization-based methods that optimize images online, and model optimization-based methods that trains the neural network for stylization. Model optimization-based methods train the network for a specific style or stylization technique. This subfield is opened by Johnson et al. [33] and Ulyanov et al. [34], that an encoder-decoder network is trained for a specific style. Huang et al. [35] introduced a method that does not require training for a specific style by using instance normalization, and improved by Li et al. [36] in their universal style transfer. More recently, Jing et al. [37] achieved great results by using dynamic instance normalization layer instead of instance normalization of [35]. Image optimization-based neural style transfer is also divided into parametric methods and non-parametric methods. Our proposed method lies in both parametric and non-parametric neural style transfer.

#### Parametric neural style transfer

Gatys et al. used Gram matrices to capture the correlations of high-level features in texture synthesis [38] and the NST [6]. Li et al. [39] proved that Gram matrix-based methods are equivalent to minimizing the Maximum Mean Discrepancy [40] using a quadratic polynomial kernel. A histogram loss was proposed by Risser et al. [13] to make NST more stable and faster. Li et al. [41] introduced a Laplacian loss between the content image and the generated image to preserve the low-level features. A video style transfer was also introduced [12, 42, 43] and expanded into [44]. Luan et al. [10] and Mechrez et al. [11] achieved photorealistic neural style transfer. Additionally, semantic-aware style transfer [45] and style transfer with controlled content features [46, 47] were introduced.

One drawback of the Gram matrix-based methods is that the slow speed of the style transfer process. To address the speed, Ulyanov et al. [34] trained a generator network and for a specific style image. Furthermore, Johnson et al. [33] trained a ConvDeconv neural network for fast style transfer as well as super-resolution. The training time and quality of results of these methods are improved in [35, 48].

#### Non-parametric neural style transfer

In contrast to the Gram matrix-based style transfer that captures features as global correlations, patch-based style transfer can capture local features. Li and Wand [14] introduced CNNMRF, a patch-based style transfer. They replaced the Gram matrix calculation with a patch-matching process performed on the outputs of a Visual Geometry Group network (VGGNet) [49]. This work has inspired research on semantic patch-based style transfer [50–52]. Furthermore, Liao et al. [53] proposed attribute style transfer. The processing time is decreased significantly by training fast semantic style transfer [54] and feed-forward networks [55, 56]. More recently, GLStyleNet [57] proposed transferring both the local features and global correlation of features.

## Guided neural style transfer for decorated shape generation

**Fig 4:**
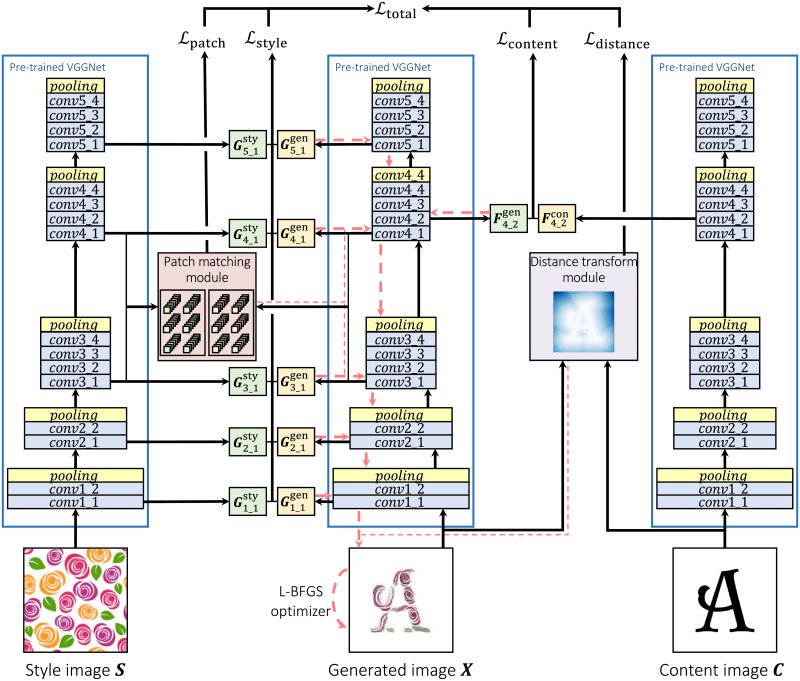
The process flow of the proposed method. Black arrows show forward operations and red arrows show back-propagation. Details of the “Distance transform module” is shown in Fig 7 and the “Patch matching module” is in Fig 8.

The proposed method is illustrated in Fig 4. In addition to capturing global features with content loss, and correlation of global features with style loss from NST [6], we used patch matching loss [14] to capture local features, and distance transfer loss [15] to constrain the style transfer process to the foreground shape. With these losses, local and global features, and correlation of global features are simultaneously transferred onto the foreground shape of the content image. In this section, we will explain the algorithm and the losses used in the proposed method.

### Neural style transfer

**Fig 5:**
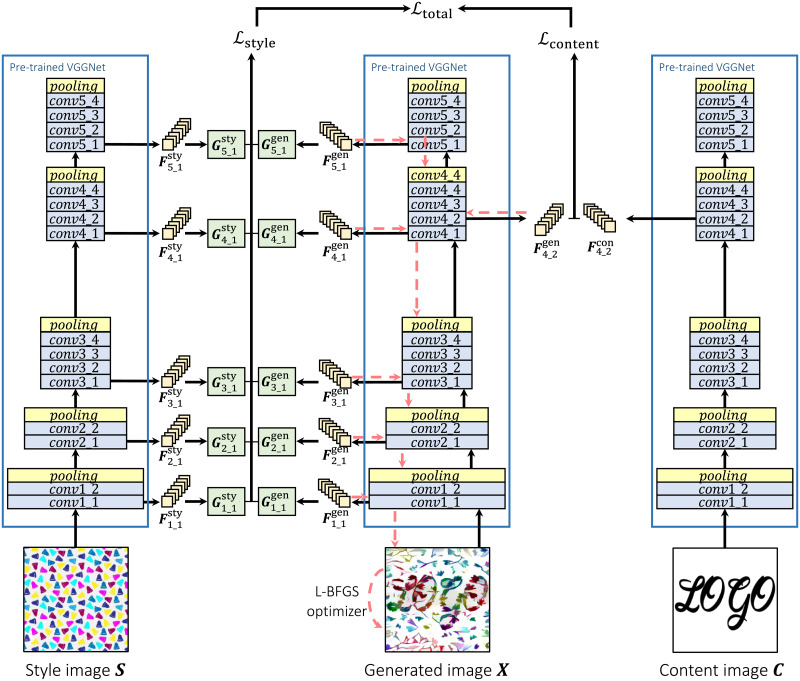
The process flow of the NST. Gram matrices are calculated from style feature maps captured by inputting style and generated images. The difference between the Gram matrices becomes style loss. Also, the difference between content features is content loss. Style and content loss are accumulated into the total loss. Using this total loss the generated image is optimized via back-propagation.

The process flow of the NST method is illustrated in Fig 5. The NST method extracts content and style representations of input images using a CNN and mixes them to optimize the generated image. A pre-trained VGGNet is used as the backbone of the method. When an image is input into the pre-trained VGGNet, it produces feature maps on every layer. The feature maps produced on the lower layers tend to represent fine details from the input image. On the other hand, the feature maps from the higher layers show arrangements of the objects inside input image. The content and style losses are computed from these feature maps for the optimization goal of the generated image.

The stylization process is as follows. Before commencing the optimization process of the generated image, content representation of the content image and style representation of the style image are calculated. First, the content image ***C*** is input into the VGGNet. Its feature maps $F^{\text{content}}$ of all layers are taken as the content representation. Then, the style image ***S*** is input into the VGGNet to determine style feature maps $F^{\text{style}}$ of all layers. Using the style feature maps $F^{\text{style}}$, Gram matrices are computed and stored as the style representation $G^{\text{style}}$ of the style image. Also, the generated image ***X*** is initialized. It can be initialized as anything, e.g. as the content image or as a random noise image.

At the beginning of the optimization process, the generated image ***X*** is input into the VGGNet. Content representation $F^{\text{generated}}$ and style representation $G^{\text{generated}}$ are extracted in the same manner as with the content and style images, respectively. Content loss $L_{\text{content}}$ is determined as a sum of the squared difference between content representations of content and generated images. Style loss $L_{\text{style}}$ is determined as a sum of the squared difference between style representations of the style and generated images. These losses are accumulated into the total loss $L_{\text{total}}$. Namely, the total loss $L_{\text{total}}$ is calculated by:

$$\begin{array}{c} L_{\text{total}}=\alpha L_{\text{content}}+\beta L_{\text{style}}, \end{array}$$ (1)

where *α* and *β* are weighting hyperparameters. With the total loss $L_{\text{total}}$, gradients of the generated image are calculated by using back-propagation through the used layers. Then the optimizer optimizes the generated image ***X*** using its updated gradients.

As mentioned before, the original NST uses only content loss $L_{\text{content}}$ and style loss $L_{\text{style}}$ to optimize the generated image. Below is a detailed explanation for content and style losses.

### Content loss

Using the feature maps of the content image $F^{\text{content}}$ and the feature maps of the generated image $F^{\text{generated}}$ as the content representations, content loss $L_{\text{content}}$ for all selected set of layers *L*_*c*_ is defined as:

$$\begin{array}{c} L_{\text{content}}=\sum_{l}^{L_{c}}\frac{w_{l}^{\text{content}}}{2N_{l}M_{l}}\sum_{n_{l}}^{N_{l}}\sum_{m_{l}}^{M_{l}}{\left(F_{l,n_{l},m_{l}}^{\text{generated}}-F_{l,n_{l},m_{l}}^{\text{content}}\right)}^{2}, \end{array}$$ (2)

where *l* refers to a layer, *N*_*l*_ is the number of feature maps, *M*_*l*_ is the number of elements in one feature map of the layer, and $w_{l}^{\text{content}}$ is a pre-defined weight for the layer. Also, each element of $F^{\text{content}}$ and $F^{\text{generated}}$ is written as $F_{n_{l},m_{l}}^{\text{content}}$ and $F_{n_{l},m_{l}}^{\text{generated}}$, respectively. Content loss preserves the general shape (content) of the content image by penalizing the change in the content representation of the generated image. Due to the higher layers learning high-level features such as the structures of the content image, it is preferable to calculate the content loss from higher layers.

### Style loss

For the calculation of the style representation, the Gram matrix is used. The Gram matrix **G**_*l*_ of layer *l* is defined as:

$$\begin{array}{c} G_{l}=S_{l}{\left(S_{l}\right)}^{⊤}, \end{array}$$ (3)

where ***S***_*l*_ is a matrix that consists of flattened feature maps from layer *l*. With *N*_*l*_ as the number of feature maps on layers *l* and ${\vec{F}}_{n_{l}}$ as an individual flattened feature map, ***S***_*l*_ can be written as follows:

$$\begin{array}{c} S_{l}=\left\{{\vec{F}}_{1},\ldots ,{\vec{F}}_{n_{l}},\ldots ,{\vec{F}}_{N_{l}}\right\}. \end{array}$$ (4)

The texture information (style) is incorporated into the Gram matrix by correlating the feature maps of a given layer. To capture consistent and scalable style representation G, Gram matrices are computed generally on layers conv1_1, conv2_1, conv3_1, conv4_1, conv5_1 of the VGGNet.

With style representation of the style image as $G^{\text{style}}$, style representation of the generated image as $G^{\text{generated}}$ and *w*_*l*_ as a pre-defined weight for a style layer *l*, style loss $L^{\text{style}}$ on all selected set of layers *L*_*s*_ is defined as,

$$\begin{array}{c} L_{\text{style}}=\sum_{l}^{L_{s}}\frac{w_{l}^{\text{style}}}{4N_{l}^{2}M_{l}^{2}}\sum_{i}^{N_{l}}\sum_{j}^{N_{l}}{\left(G_{l,i,j}^{\text{generated}}-G_{l,i,j}^{\text{style}}\right)}^{2}. \end{array}$$ (5)

In Eq 5, $G_{l,i,j}^{\text{generated}}$ and $G_{l,i,j}^{\text{style}}$ refers to the values at indices (*i*, *j*) of the Gram matrices on layer *l*. Style loss ensures that the correlation of global features (style) are the same on both the style and generated images. On every block of layers, style loss tends to capture different features; therefore, the layers from each block are used as the style layers.

### Distance transform loss

**Fig 6:**
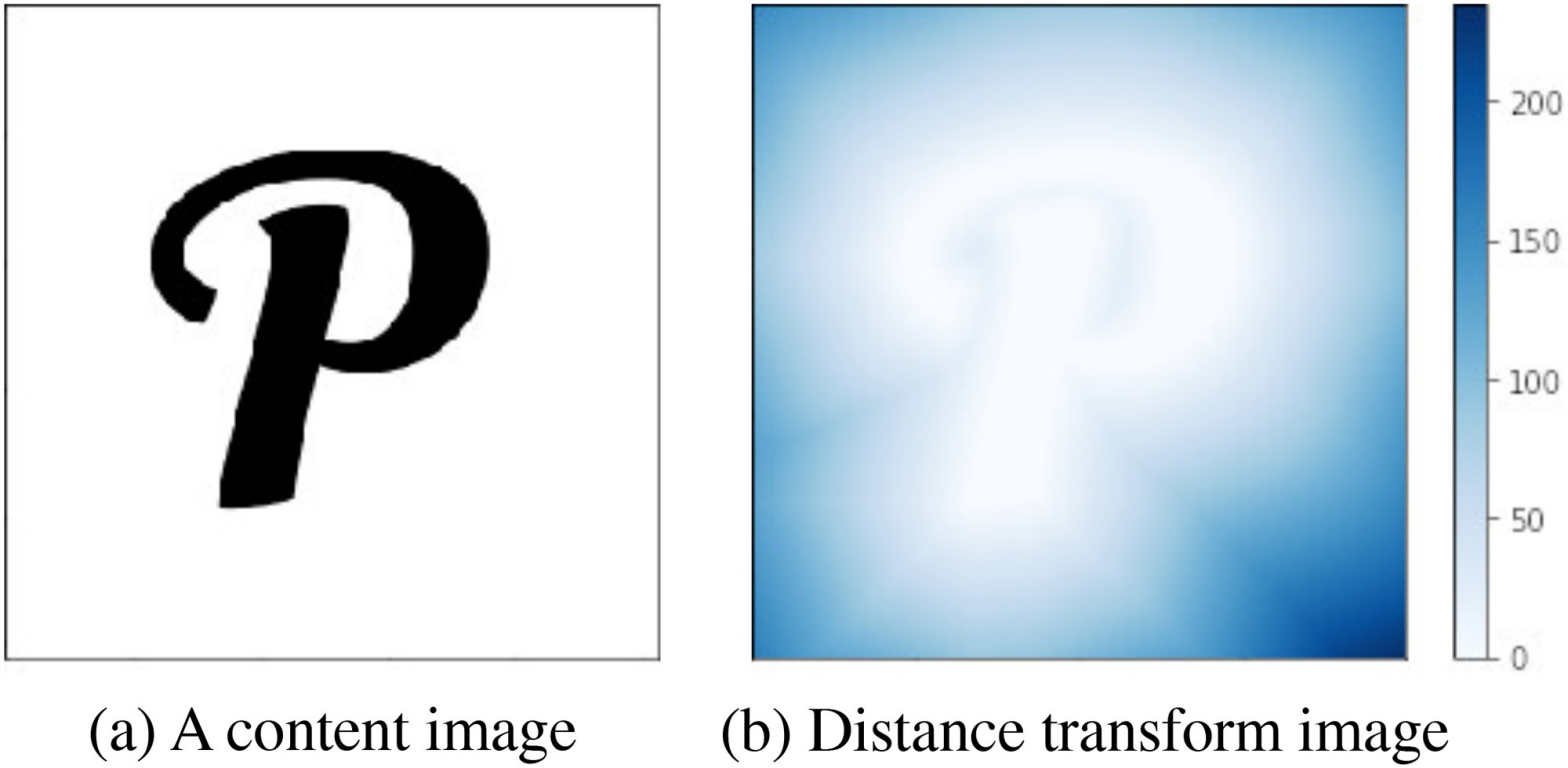
An example of an image with its respective distance transform.

Unlike natural images, shapes are typically isolated structures with plain backgrounds, as shown in the content image of Fig 6. Consequently, standard NST methods will place unwanted style features in the background area. To address this problem, Atarsaikhan et al. [15] introduced Distance Transform Loss that is calculated from the distance transform of the content image. The distance transform assigns a value for each pixel of a binary image based on the distance to the nearest foreground pixel. Therefore, the binary image has a pixel value of 0 on the foreground shape and pixel value gradually increases based on the distance from the shape. An example of distance transform is show in Fig 6. Furthermore, the distance transform **D** of content image ***C*** can be tuned to emphasize the distance between the foreground and the background using the power *z*.

**Fig 7:**
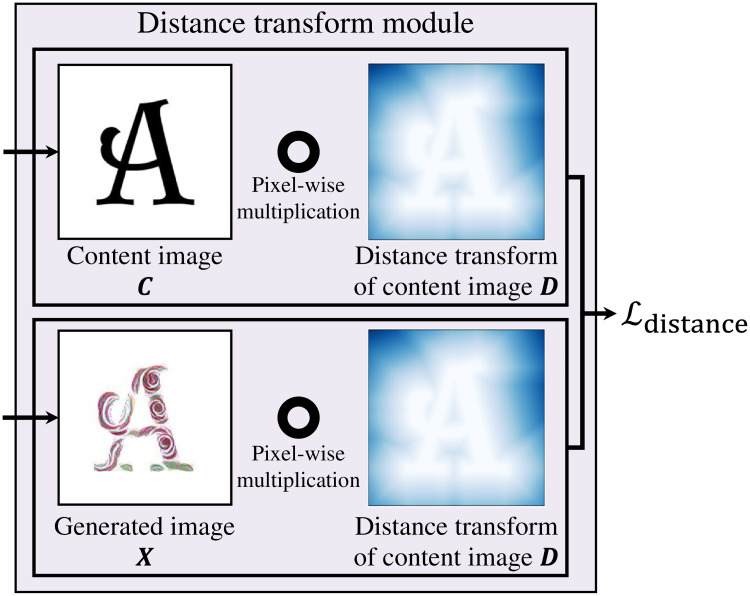
Distance transform module. Distance transform loss $L_{\text{distance}}$ is calculated as, the difference between pixel-wise multiplication of the content image ***C*** and **D**, and pixel-wise multiplication of the generated image ***X*** and **D**.

Using generated image ***X***, content image ***C***, and emphasized distance transform **D**^*z*^ of the content image, distance transform loss $L_{\text{distance}}$ is defined as,

$$\begin{array}{c} L_{\text{distance}}=\frac{1}{2}∥C\circ D^{z}-X\circ D^{z}∥, \end{array}$$ (6)

where ∘ stands for pixel-wise multiplication. This process is shown in distance transform module in Fig 7. The biggest advantage of utilizing distance transform loss is that it constrains stylization to the foreground shape while permitting soft transformations near the edge of the foreground. Distance transform loss ensures that the continuity of the style around the foreground edges is preserved without any abrupt cuts. In other words, it performs a soft masking operation, instead of the regular hard masking.

### Patch matching loss

Visually plausible results can be achieved using content loss to preserve the shape of the content image, style loss to transfer the style of the style image, and distance transform loss to constrain the transfer to the content image. However, due to the use of a Gram matrix between feature maps, the results are unstable and hard to control. The Gram matrix statistically abbreviates feature maps of the style image and tries to match with the Gram matrix of the generated image. However, there can be more than one set of feature maps producing the same Gram matrix. Thus, the Gram matrices from the style image and the generated image can be the same, even if their styles are not the same. Moreover, the Gram matrix is designed to capture correlations of global features and ignore the local features.

Li et al. [14] proposed a patch matching technique to extract styles that include local features. They divided the feature maps from the VGGNet into small patches, and the patches from style image and generated image are matched to achieve style transfer. This technique ensures that the feature maps of the style image and the generated images are similar. However, the problem with this method is that it only produces attractive results if the content and style images are already quite similar. Intuitively, patch-based style transfer methods lack the abstract abilities of the standard NST using the Gram matrices.

We combined the advantages of these two methods by using both style loss and a patch matching loss which is inspired from [14]. When an image is input into a VGGNet, patches are extracted from feature maps on specified patch layers. The size of one patch is [*C* × *k* × *k*] with stride = 1, where *k* is height and width and *C* as the number of nodes in the layer. We extract patches from conv3_1 and conv4_1, because these layers capture both local and global features in contrast to lower layers that capture colors and borders, or higher layers that capture global features only. Given style image ***S*** and generated image ***X***, we extracted style patches as $P^{\text{style}}$ and generated patches as $P^{\text{generated}}$, respectively.

**Fig 8:**
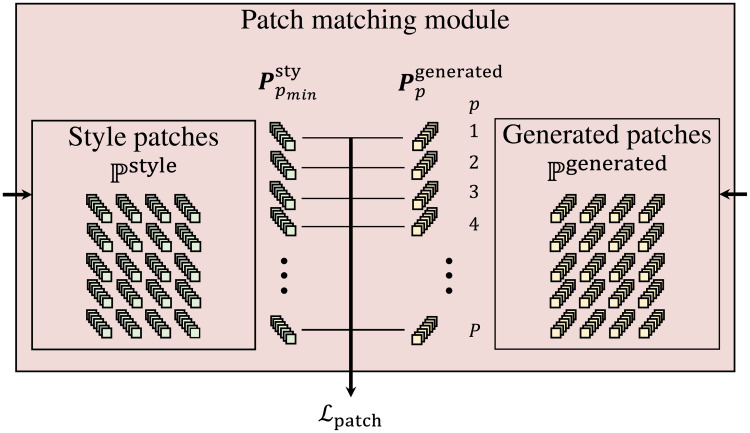
Patch matching module. Patches are extracted as a set of generated patches $P^{\text{generated}}$ and a set of style patches $P^{\text{style}}$ from layers conv3_1 and conv4_1 of $F^{\text{generated}}$ and $F^{\text{style}}$, respectively. Because we extract patches from the same layers for generated and style images, the patch number is the same, *P*. Then, for every generated patch $P_{p}^{\text{generated}}$ with *p* as index, closest style patch $P_{p_{min}}^{\text{style}}$ with index *p*_*min*_ is found. Patch matching loss $L_{\text{patch}}$ is sum of losses on every pair of $P_{p}^{\text{generated}}$ and $P_{p_{min}}^{\text{style}}$.

The process of calculating patch matching loss is shown in Fig 8. For every generated patch ***P***^generated^ of generated patches $P^{\text{generated}}$, the best matching patch can be found from $P^{\text{style}}$ by using:

$$\begin{array}{c} p_{min}=\underset{p^{\prime }=1\ldots P}{\text{arg}\;\text{min}}\frac{P_{p}^{\text{generated}}\cdot P_{p^{\prime }}^{\text{style}}}{|P_{p}^{\text{generated}}\left|\cdot \right|P_{p^{\prime }}^{\text{style}}|}, \end{array}$$ (7)

where *p*^′^ is index and *P* is all number of patches in $P^{\text{style}}$. Also, *p* is index for patches in $P^{\text{generated}}$ and *p*_*min*_ is the closest patch index from $P^{\text{style}}$. Then, patch matching loss $L_{\text{patch}}$ is defined as a sum of the difference between generated patches and corresponding best matching patches:

$$\begin{array}{c} L_{\text{patch}}=\sum_{p}^{P}∥P_{p}^{\text{generated}}-P_{p_{min}}^{\text{style}}∥. \end{array}$$ (8)

### Style transfer process

The entire style transfer process of the proposed method is shown in Fig 4. We calculate and store content representation of content image ***C*** and style representation of style image ***S*** as previously explained. Also, style patches of style image are extracted as $P^{\text{style}}$ from patch layers. Next, generated image ***X***, which is initialized as either content image ***C*** or a random noise image, is input to the network. Content and style representations are calculated, as well as generated patches are extracted. By using content loss $L_{\text{content}}$, style loss *L*_style_, distance transform loss $L_{\text{distance}}$ and patch matching loss $L_{\text{patch}}$, the total loss $L_{\text{total}}$ can be determined as,

$$\begin{array}{c} L_{\text{total}}=\alpha L_{\text{content}}+\beta L_{\text{style}}+\gamma L_{\text{patch}}+\delta L_{\text{distance}}, \end{array}$$ (9)

where *α*, *β*, *γ* and *δ* are weighting factors for each loss. Once the total loss $L_{total}$ is determined, the generated image ***X*** is optimized by back-propagation with the goal of minimizing the total loss $L_{total}$. By repeatedly running this process, the content of the content image and the style of the style image are gradually synthesized into the generated image ***X***.

The processing time of the style transfer process depends on image size and the number of patches. The larger image size simply increases the number of nodes that had to be processed. Although larger images take longer time to stylize, the results do not necessarily become more plausible. Table 1 shows the average computation time of individual loss functions in one iteration. The patch matching loss requires the longest time to compute. Using larger patch size is more time consuming than using a smaller patch size because the number of nodes is increased with the patch size. Moreover, using stride higher than 1 significantly decreases the computation time because of less number of patches. Other losses require a small amount of time to compute however, it increased with image size. Furthermore, the initialization of the generated image affects the overall processing time. More iterations are needed to achieve the same results with random noise initialization than initializing as the content image.

**Table 1:** The average computation time of each loss in one iteration.

Image size	Patch size	Stride	Patch matching loss (ms)	Content loss & Style loss (ms)	Distance transform loss (ms)
256 x 256	5	1	380.0	4.1	0.2
		2	191.9		
	11	1	450.6		
		2	151.8		
512 x 512	5	1	2155.6	13.7	1.7
		2	445.9		
	11	1	7987.9		
		2	520.3		

## Results and discussion

**Fig 9:**
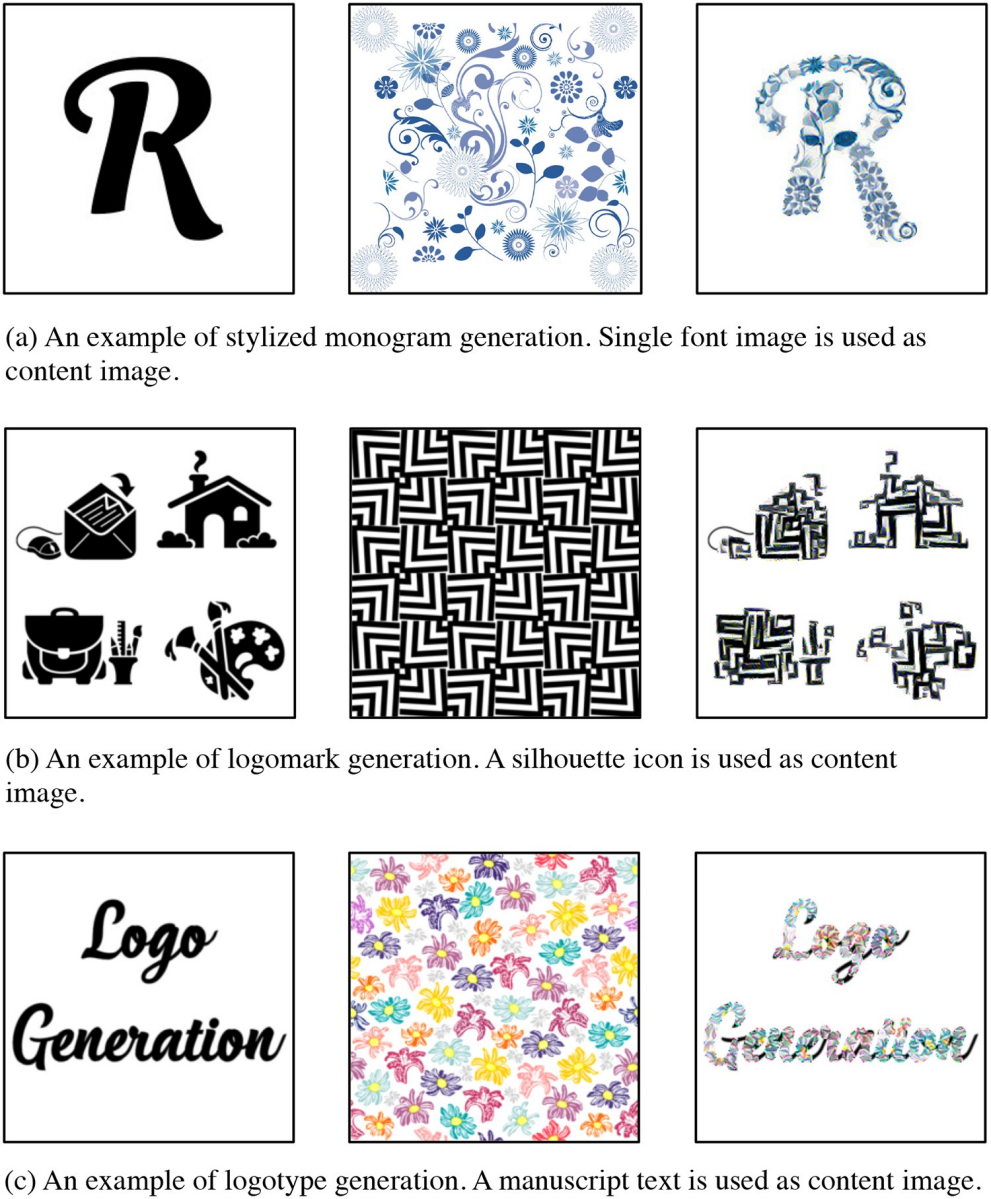
Examples of logo generation using the proposed method.

We used silhouette icons, single letter images, and texts as content images. Examples of the stylized shapes using the proposed method are shown in Fig 9. In Fig 9A, a monogram is generated from a single “R” image and style image. Fig 9B shows a logomark that is created from silhouette icons and Fig 9C has a text as logotype.

### Qualitative evaluation

To analyze the capability and quality of the proposed method, we performed the following comparison experiments:

- *NST* [6]. The standard NST method which uses only style loss $L_{\text{style}}$ and content loss $L_{\text{content}}$. NST is run for 5,000 iterations.
- *Constrained NST* [15]. NST using style loss $L_{\text{style}}$ and content loss $L_{\text{content}}$ plus the additional distance transform loss $L_{\text{distance}}$. Constrained NST is also run for 5,000 iterations.
- *CNNMRF* [14]. This comparison is the model proposed by Li et al. which introduced patch matching based NST. Only patch matching and the patch matching-based loss $L_{\text{patch}}$ is used. This network is run for 2,000 iterations.
- *NST + Patch matching*. This evaluation is to demonstrate the effects of using NST with only style loss $L_{\text{style}}$, content loss $L_{\text{content}}$, and the patch matching loss $L_{\text{patch}}$. Using NST + Patch matching is run for 2,000 iterations.
- *Arbitrary Style Transfer in Real-time with Adaptive Instance Normalization (AdaIN)* [35]. Huang et al. stylize contents by using instance normalization. It is possible to perform spatial control by masking feature maps on each layer.
- *Universal Style Transfer via Feature Transforms (UST)* [36]. Although similar to AdaIN, UST uses whitening and coloring transformation instead of instance normalization. As for spatial control, it can be achieved by masking feature maps on each layer.
- *Proposed method*. This is the proposed method of using all four losses and is run for 2,000 iterations.

In the experiments, all image sizes are 256 × 256. The pre-defined weights for content layers are zero except for $w_{4}^{\text{content}}\_2=1$, and weights for style layers are zero except for $w_{1}^{\text{style}}\_1={\left(\frac{10}{64}\right)}^{3}$, $w_{2}^{\text{style}}\_1={\left(\frac{10}{128}\right)}^{3}$, $w_{3}^{\text{style}}\_1={\left(\frac{10}{256}\right)}^{3}$, $w_{4}^{\text{style}}\_1={\left(\frac{10}{512}\right)}^{3}$, $w_{5}^{\text{style}}\_1={\left(\frac{10}{512}\right)}^{3}$. For the methods that use patch matching, patch sizes are *C* × 5 × 5 and are taken from the layers conv3_1, and conv4_1. The weighting factors for each loss are *α* = 0.1, *β* = 0.1, *γ* = 10^−6^ and *δ* = 100. All of the experiments are performed on single “GeForce GTX 1080 Ti” GPU.

**Fig 10:**
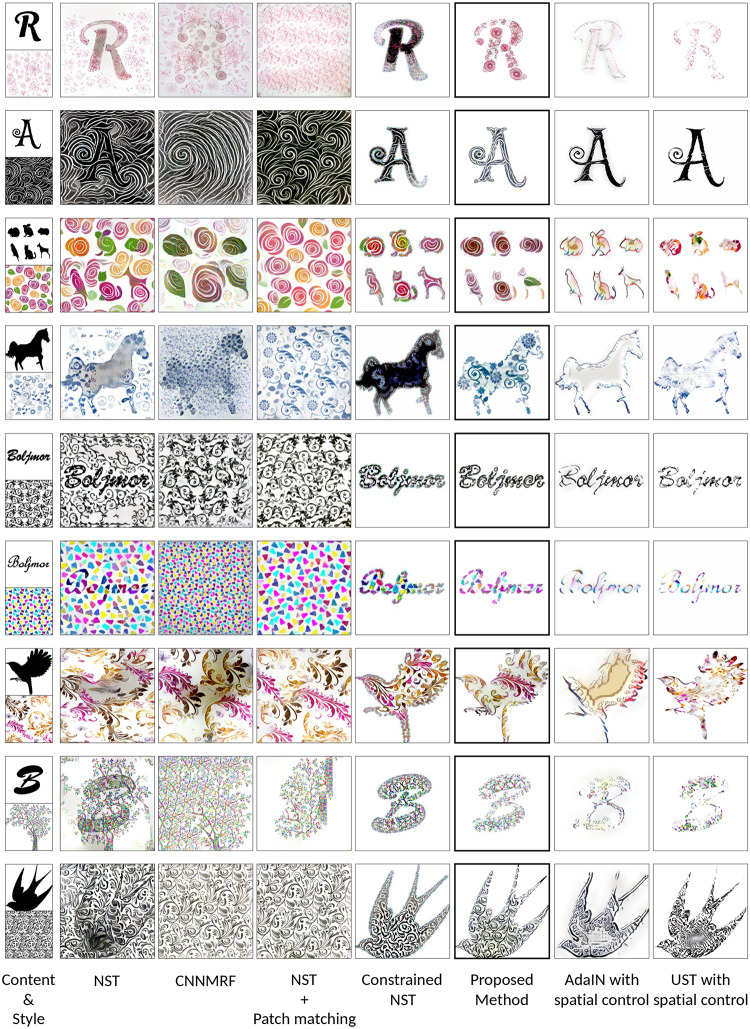
The results comparison with NST, CNNMRF, Constrained NST, NST + Patch matching, AdaIN with spatial control, and UST with spatial control methods. The first column shows various content and style combinations, and each column shows results from each method using the content and style combination from the left.

In Fig 10, we compare the results of the above methods. Due to the lack of constraint from a distance transform loss, NST, CNNMRF, and NST + Patch matching have unnecessary background noise. Although, in the NST results, the contents of the content image are more visible than CNNMRF. This is because, in NST, feature maps are abstracted with a Gram matrix and do not require to look exactly like the style image. In CNNMRF, patches from feature maps are directly matched, thus, the method neurally copies and pastes style features. NST + Patch matching method transfers local features as well as correlation of global features from the style image. Thus, the results are visually comparable to the style image. However, these methods do not have constrain or control to ignore the background of the content image. So, there are styles on all of the generated images, foreground and background indiscriminately.

Conversely, using the NST with distance transform loss $L_{\text{distance}}$ in the Constrained NST, the style transfer is contained in the shape correctly. However, because the content images and the style images are not similar enough, the results of using just a Gram matrix for style transfer are not sufficiently impressive. There are areas that the Constrained NST could not transform and corrupted colors in the logotype results.

The spatial controls in AdaIN and UST worked fairly well. The results are appealing, and there is no style on the background. However, details (e.g. round shapes, patterns) of the style image are completely ignored. Instead, new details with color similar to the style image emerged. The biggest advantage of these methods is the feed-forward stylization. Because of that, they are generally much faster than the proposed method.

On the other hand, results from the proposed method are not only constrained to the shape of the content image but also included the details from style image. Distance transform loss $L_{\text{distance}}$ constrains the style transfer and the patch matching loss $L_{\text{patch}}$ ensures that there is no corrupted colors or different details. The results in Fig 10 demonstrate that the proposed method combines the benefits of patch matching and the distance constraint. Similar to the CNNMRF and NST + Patch matching, the proposed method has detailed local features, and similar to Constrained NST, the proposed method does not have an extra background style.

### On simple masking

**Fig 11:**
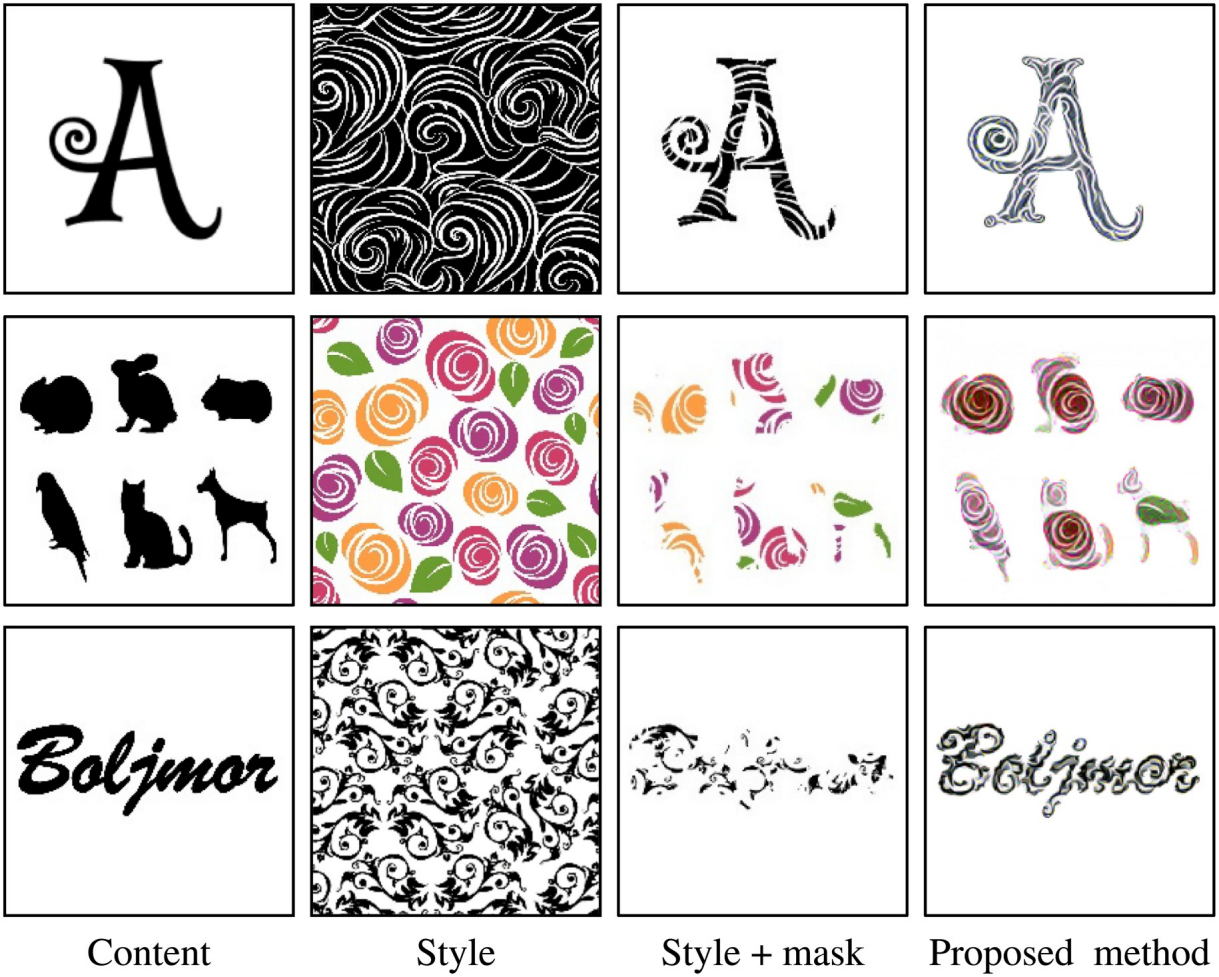
Comparison of the proposed method against masking the style image.

One concern would be that simply masking the style image would produce better results. However, Fig 11 shows that a simple masking operation does a poor job. The results from the masking operation have unnatural cuts of the style, whereas the results from the proposed method preserved the style as natural as possible. Specifically, as shown in the first row, the style is black areas, and thin white stripes. By masking this style with the content letter of “A”, white stripes are cut in the middle. However, the result from the proposed method has continual white stripes. In rows 2 and 3, the masking operation resulted in incoherent contents. This shows the results of the proposed method is better than a simple masking operation.

### Ablation study

In this section, we discuss the effects of some of the hyperparameters of the proposed method.

#### Effects of weighting factors

**Fig 12:**
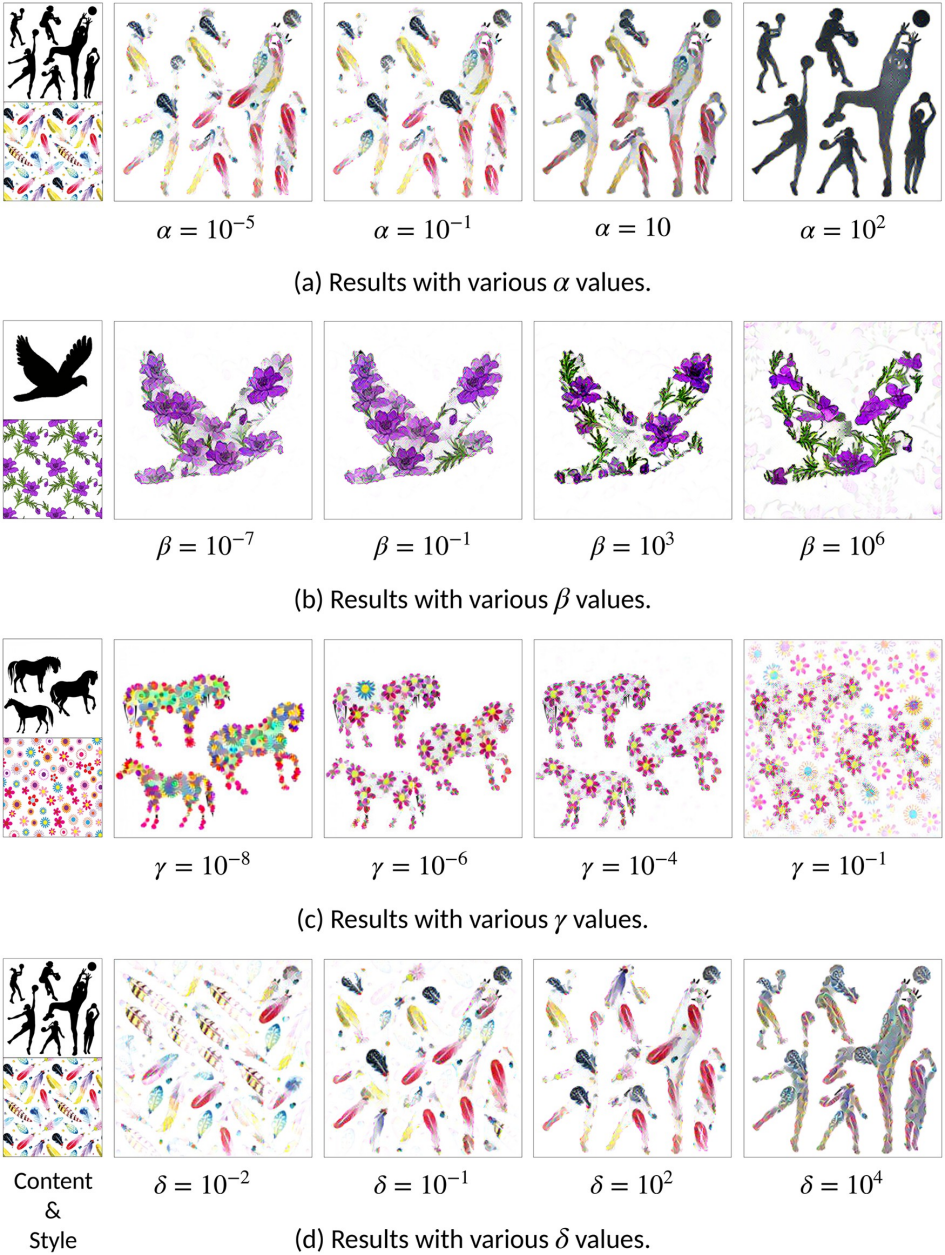
Style transferring results with various parameters.

In Eq (9), the different losses are weighted respectively. Different weighting factors have different effects on the results. Hence, it is important to understand the effect of weighting factors. Fig 12 shows the effects of each weighting factor while other weighting factors are fixed. The magnitude of the weighting factor is attributable to the magnitude of the respective loss, in comparison to the other losses. In Fig 12A, there are results with various *α* for content loss. If *α* is larger the style transfer process is hindered, and the results are more similar to the content image. However, if *α* is smaller, content shapes could not be recognized from the results. Fig 12B shows various *beta* for style loss. With smaller *β* local features are dominant, while with bigger *β* style loss neglected other losses, especially distance transform loss. Distance transform loss could be overwhelmed by patch matching loss with large *γ* as shown in Fig 12C. Smaller *γ* means that the global feature is confined into the shape of the content image. Moreover, distance transform loss with small *δ* could not be able to constrain the style transfer process. On the other hand, if the *δ* is too large, it hindered the style transfer process. There is a trade-off between these weighting factors, and we found that *α* = 0.1, *β* = 0.1, *γ* = 10^−6^ and *δ* = 100 work best.

#### Patch size

**Fig 13:**
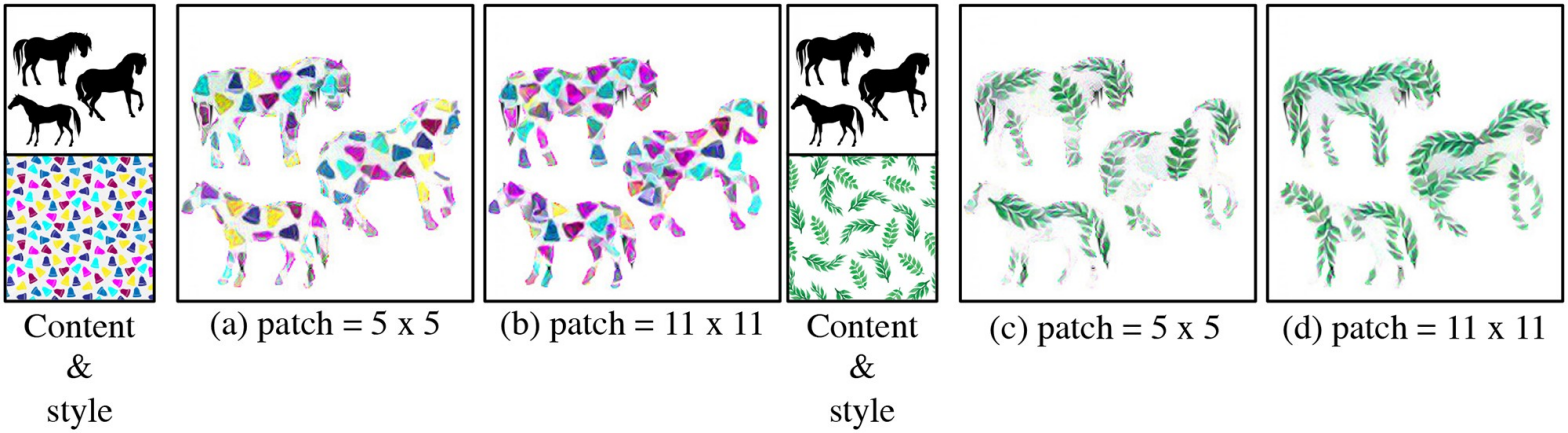
The effects of patch size that used in the proposed method.

The population of the style patches in the generated image can be controlled by changing patch size. Fig 13 shows experimental results on the patch sizes of patch matching module. With smaller patches, such as 5 × 5, more densely populated styles can fit into the shape as shown in Fig 13A and 13C. On the other hand, as shown in Fig 13B and 13D, with larger patches, like 11 × 11 the styles are scarce. The reason for that is when the patch size is small, it includes the part of feature maps that represents either of the style or the background, separately. Then, in the patch matching process, the patches that include only background features are matched to the background of the content image and patches of the foreground are matched properly. With large patches like 11 × 11, the windows are so large that they include both the foreground and background. When applied to the generated image, excess background from the style image appears.

### Limitations

**Fig 14:**
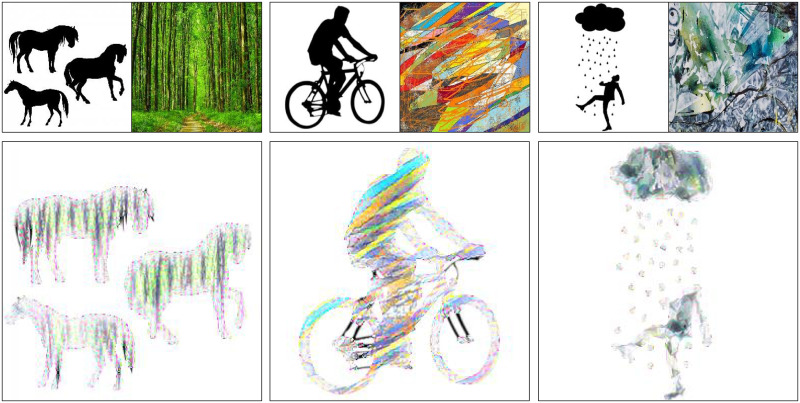
Photos and images without plain background as style images.

**Fig 15:**
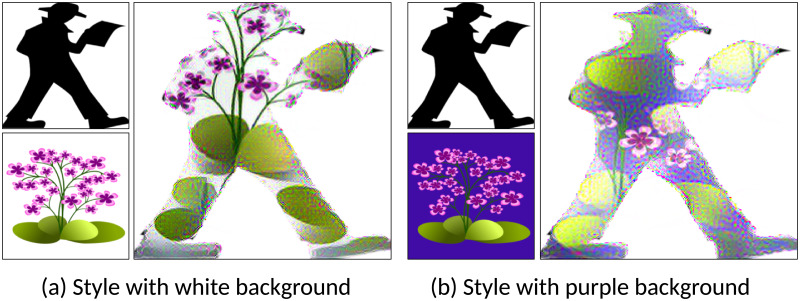
White background style image vs colored background image.

While the proposed method produces generally good results, depending on the choice of the content and style image combinations the quality of the generated images can vary. In Fig 14, style images have no specific background. Although some features of the style images were transferred onto the shape, the stylized results are not as plausible as other results. That is because the proposed method tried to generate all of the style image onto the shape of content image, which is too small for the entire style image. Fig 15 compares style images with white background and purple background. The user has to specify the background color of the input images. In this paper, we specified the background color as white because most logo images have a white background. So, when a style image with a white background is used, the white background was rejected in the patch matching process. Thus, the results have only flowers and green leaves. However, when a purple background style image is used, the proposed method cannot discriminate between the background. Hence, the generated image has all features from the style image; flowers, green leaves, and purple background.

**Fig 16:**
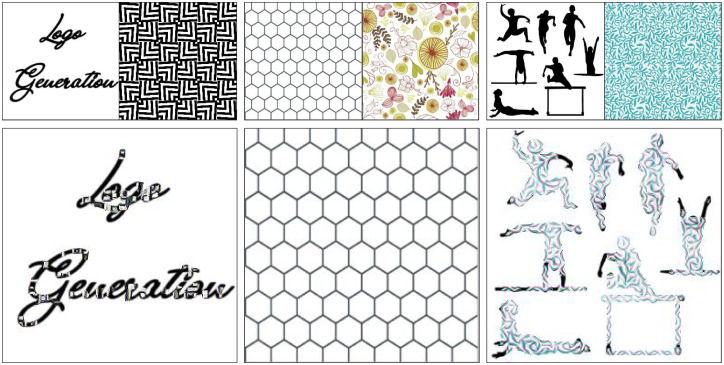
Too narrow content images.

Moreover, Fig 16 shows results that are generated from content images that have too narrow shapes compared with the style. The first image shows that when the content shape is too narrow, there is not enough room for the style. Thus, the results change very little, or in extreme cases does not change at all, as shown in the second image. Furthermore, the third image shows that there is no change in the narrow part of the content image whereas the wider parts are stylized.

**Fig 17:**
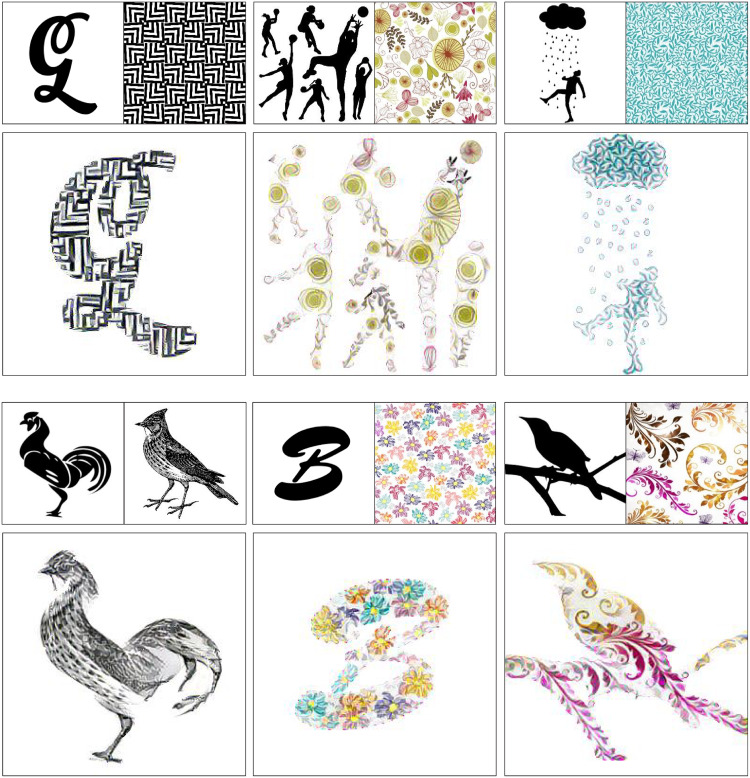
Examples of good combinations of content and style images. Content and style combinations that produce good results. The upper smaller images are the content and style images, respectively. The larger images are the generated results.

Alternatively, Fig 17 shows various combinations of content images and style images that produce good results. With the same size objects, the style transfers much more easily because of the patch matching module and Gram matrix style transfer. We also found that style images with many different but somewhat repetitive features work well. This is due to the global correlation of features in the Gram matrix. In addition, it is better to have many instances of similar but slightly different features. Finally, the objects inside the content images should be large enough to be able to contain multiple features from the style image. This also enables them to be redrawn by the style from the style image.

Table 2 shows the overall computation time of each method with various parameters. The proposed method is much slower than other methods. Due to the high number of nodes in the extracted patches, the backward calculation of gradients with regard to the total loss consumes a large amount of time. One solution is to use a larger stride. With a larger stride, the number of nodes can be decreased significantly and make the proposed method faster. NST and Constrained NST have similar computation time because distance transform loss requires a negligible amount of computation time. CNNMRF is also fast because it resizes the image to a smaller size and gradually increases the image size while stylizing. AdaIN and UST require one forward-pass for stylization, however, prior training is also needed.

**Table 2:** Computation time of various methods.

Methods	Image size	Patch size	Number of iterations	Computation time (min)
NST	256 x 256	-	5000	2.10
	512 x 512			5.33
Constrained NST	256 x 256			2.01
	512 x 512			5.60
CNNMRF	256 x 256	5	2000	2.04
		11		1.29
	512 x 512	11		14.15
		5		4.60
**Proposed method**	512 x 512	5		33.56
		11		32.81
	256 x 256	5		360.63
		11		467.91
AdaIN	512 x 512	-	1	3.59 sec
	256 x 256			3.68 sec
UST	256 x 256			5.14 sec
	512 x 512			5.9 sec

## Conclusion

In this paper, we integrated a patch matching process into the Gram matrix-based style transfer method for stylizing shapes by transferring local and global correlation of features from a style image. Using a patch matching loss, we were able to transfer the local features that lack in the regular NST. Then, we constrained the style transfer process only to the contents of the content image using a distance transform loss. Compared to hard cutting, such as masking, the proposed method ensures continuity of the transferred styles in the generated image. Through experimental results, we demonstrated that the proposed method outperformed multiple style transfer methods such as NST, CNNMRF, NST + Patch matching, Constrained NST, AdaIn, UST, and simple masking. Furthermore, we discussed the effects of different hyperparameters and different content and style images on the generated results. We demonstrated that the proposed method can generate attractive shapes easily without requiring prior training and manual instructions for the style.
